# Cuidados Paliativos na Insuficiência Cardíaca Descompensada com Necessidade de Terapia Inotrópica: Oportunidades de Integração para Melhoria dos Desfechos

**DOI:** 10.36660/abc.20250188

**Published:** 2026-01-09

**Authors:** Daniel Battacini Dei Santi, Mucio Tavares de Oliveira, Ricardo Tavares de Carvalho

**Affiliations:** 1 Instituto do Coração do Hospital das Clínicas Faculdade de Medicina Universidade de São Paulo São Paulo SP Brasil Instituto do Coração do Hospital das Clínicas da Faculdade de Medicina da Universidade de São Paulo, São Paulo, SP – Brasil; 2 Hospital das Clinicas Faculdade de Medicina Universidade de São Paulo São Paulo SP Brasil Hospital das Clinicas da Faculdade de Medicina da Universidade de São Paulo – Cuidados Paliativos, São Paulo, SP – Brasil

**Keywords:** Insuficiência Cardíaca, Choque Cardiogênico, Cuidados Paliativos, Estado Terminal, Cuidados Paliativos na Terminalidade da Vida

## Abstract

**Fundamento:**

A insuficiência cardíaca (IC) avançada apresenta elevada morbidade e mortalidade, comprometendo a funcionalidade e a qualidade de vida de pacientes e familiares. Hospitalizações elevam a gravidade, especialmente quando requerem terapia inotrópica. Os cuidados paliativos (CP) auxiliam a lidar com o sofrimento causado por doenças graves, mas são pouco realizados na cardiologia.

**Objetivo:**

avaliar a integração dos CP na abordagem de pacientes com IC descompensada, identificando possibilidades de melhoria assistencial.

**Método:**

estudo unicêntrico, retrospectivo, observacional, realizado entre fevereiro/2015 e maio/2018, com pacientes com IC em terapia inotrópica. Analisaram-se os encaminhamentos para CP, a abordagem da equipe de interconsulta e os desfechos, com análise de sobrevida em 5 anos. Nível de significância estatística: 5%.

**Resultados:**

Foram incluídos 492 pacientes, 66,9% do gênero masculino, mediana de idade de 63 anos (IQ 52-72). O referenciamento para CP ocorreu em 23% da amostra, com mediana de 8,0 dias (IQ 4,0-20) antes do óbito. Somente 14% dos pacientes em terapia intensiva foram referenciados. Nenhum paciente transplantado recebeu assistência paliativa. Pacientes atendidos pela equipe de CP foram mais envolvidos na tomada de decisão e mais frequentemente receberam opioides para manejo de sintomas do que os atendidos exclusivamente por cardiologistas (p<0,01). A mortalidade hospitalar e em 5 anos foi de 42% e 80%, respectivamente.

**Conclusões:**

Pacientes em descompensação de IC apresentam alta mortalidade e são pouco encaminhados para CP, geralmente nos últimos dias de vida, o que reduz os benefícios dessa abordagem. É necessário maior educação médica em CP e o desenvolvimento de estratégias para aumentar essa integração, o que pode resultar em melhores desfechos.

## Graphical abstract

**Figura Central:**
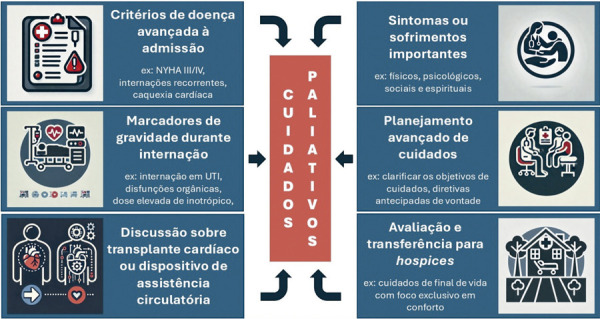
Cuidados Paliativos na Insuficiência Cardíaca Descompensada com Necessidade de Terapia Inotrópica: Oportunidades de Integração para Melhoria dos Desfechos

Oportunidades de integração de cuidados paliativos em pacientes com choque cardiogênico.

## Introdução

A insuficiência cardíaca (IC) apresenta elevada mortalidade no mundo todo, sendo responsável por mais de 9 milhões de óbitos todos os anos.^1^ No Brasil, se estima um acometimento de 2 milhões de pessoas, com registro de mais de 240 mil novos casos anualmente.^2^ Em 2019, houve mais de 196 mil internações por IC, sendo que os gastos em serviços de saúde com essa condição ultrapassam os R$ 3 bilhões.^3^

A hospitalização ocorre quando há piora dos sintomas, o que aumenta os riscos, principalmente naqueles que apresentam sintomas congestivos e de baixo débito cardíaco, classificados como “perfil clínico hemodinâmico C”, conhecido como “frio e úmido”.^4-6^ Há maior demanda de suporte hospitalar, com internações mais prolongadas e maior utilização de recursos para suporte de vida.^5,6^

As terapias modificadoras de doença para pacientes com IC avançada são limitadas a poucas estratégias, como o transplante cardíaco e dispositivos de assistência circulatória mecânica (DACM), que são disponíveis apenas para uma pequena parcela dos pacientes.^7,8^ Estes pacientes experienciam sintomas importantes, que limitam a sua funcionalidade e qualidade de vida, se fazendo necessário a realização de abordagens complementares ao tratamento convencional.^7,8^

Os cuidados paliativos (CP) têm um papel de destaque nesse contexto.^9,10^ Radbruch et al. os definem como um “cuidado holístico ativo de indivíduos de todas as idades com sofrimento significativo relacionado à saúde devido a doenças graves, especialmente daqueles em fase final de vida. O objetivo é melhorar a qualidade de vida dos pacientes, de suas famílias e de seus cuidadores.”^11^

Nos últimos anos as diretrizes de sociedades de especialistas têm recomendado a integração precoce dos CP no tratamento de pacientes com IC,^12-14^ principalmente para auxiliar no manejo de sintomas e no planejamento terapêutico.^9,11,15,16^ Essa abordagem pode influenciar positivamente nos desfechos clínicos, como evitar intervenções fúteis e favorecer a transferência para unidades *hospices*, que são estabelecimentos focados em oferecer conforto e atender as necessidades individuais no final de vida.^9,10,17-19^

No entanto, há diversas barreiras à integração dos CP na cardiologia, como a falta de conhecimento dos profissionais de saúde e a percepção equivocada de que os CP são destinados a pacientes em fim de vida, no contexto de ausência de perspectivas terapêuticas modificadoras para a doença.^20,21^ A imprevisibilidade da trajetória clínica da IC, a dificuldade de estabelecer um prognóstico preciso e a falta de padronização de critérios de referenciamento, são fatores que dificultam essa integração.^22^ A mudança desse cenário requer medidas estruturais, como a ampliação da educação médica, a criação de protocolos assistenciais e o fortalecimento de incentivos institucionais, promovendo uma transformação cultural que melhore a qualidade da assistência.

Pacientes em descompensação de IC com necessidade de terapia inotrópica são pouco estudados na literatura, principalmente com relação à abordagem de CP.^23^ Este estudo tem o objetivo de investigar como é feita a integração de especialistas em CP no tratamento a estes pacientes, explorando oportunidades para otimizar a assistência.

## Métodos

### Desenho do estudo e população

Este é um estudo observacional, retrospectivo, unicêntrico, realizado em um hospital escola quaternário brasileiro, referência para o tratamento de paciente com IC. A coorte foi composta por pacientes internados por descompensação de IC em perfil C, com necessidade de terapia inotrópica, admitidos no pronto-socorro entre 01 de fevereiro de 2015 e 31 de maio de 2018.

Os pacientes foram selecionados a partir dos registros de internação com CID-10 de IC (I.50), sendo seus dados coletados através dos registros eletrônicos hospitalares, com transcrição para uma planilha de dados protegida, para posterior análise estatística. A sobrevida da amostra foi analisada em 5 anos.

Foram incluídos pacientes com diagnóstico prévio de IC, independentemente da etiologia, com documentação ao ecocardiograma de fração de ejeção de ventrículo esquerdo menor que 50% ou disfunção moderada ou importante de ventrículo direito. Os pacientes deviam apresentar critérios clínicos ou laboratoriais que permitissem a sua classificação em perfil C e a terapia inotrópica ter sido iniciada nos primeiros 5 dias da admissão hospitalar.

Foram excluídos da amostra pacientes menores de 18 anos, com diagnóstico de IC “nova” ou nos quais a síndrome coronariana aguda fosse a principal hipótese diagnóstica. Também foram excluídos pacientes caso o inotrópico tivesse sido utilizado por menos de 24 horas ou em decorrência de intervenção anestésico/cirúrgica. Pacientes transferidos de outros serviços em uso de inotrópicos ou vasopressores, ou cujos registros eletrônicos iniciais não permitissem a caracterização da admissão hospitalar, ou aqueles em que o término da internação ocorreu fora do complexo hospitalar, também foram excluídos da amostra. Em caso de mais de uma admissão no pronto-socorro no período do estudo, foi considerada apenas a primeira internação que preenchesse esses critérios.

**Variáveis de interesse:**

Foram analisadas as características clínicas e demográficas dos pacientes e características da admissão no pronto-socorro, com análise do risco realizada através da ferramenta *Acute Decompensated Heart Failure National Registry* (ADHERE).^24^ Analisou-se também o período de internação, os tratamentos realizados e como foi feito o referenciamento para a equipe de CP.

Essa investigação avaliou a frequência de solicitações de interconsulta e o tempo necessário para o encaminhamento, as características dos pacientes encaminhados, os contextos de internação em que foram feitas as solicitações de interconsulta e as intervenções realizadas pela equipe de interconsulta de CP.

Os pacientes que foram atendidos pela equipe de CP foram comparados com aqueles acompanhados exclusivamente por cardiologistas quanto ao uso de opioide no final de vida, participação dos pacientes nas discussões de tomada de decisão no final de vida e encaminhamento para o ambulatório de CP após a alta.

### Análise estatística

Os dados foram descritos em frequências absolutas e relativas. Para comparação das solicitações de interconsulta ao longo dos 40 meses do estudo, foram discriminados quatro intervalos de 10 meses. Variáveis contínuas foram apresentadas em mediana e intervalos interquartis (IQ) de 25% e 75%, e as comparações entre grupos foram realizadas através do teste qui-quadrado. Para estimar a razão de prevalência não ajustada e ajustada para os desfechos binários de interesse, foi utilizado o modelo de regressão de Poisson.

As variáveis utilizadas na regressão multivariada foram: idade, fração de ejeção, internação hospitalar nos últimos 12 meses, classe funcional *New York Heart Association* (NYHA), uso prévio de terapia medicamentosa direcionada por diretrizes (TMDD), insuficiência renal, parada cardiorrespiratória, terapia inotrópica (tempo de utilização, necessidade de combinação de inotrópicos ou de dose máxima) e propostas terapêuticas para IC avançada durante a internação.

A análise da probabilidade de sobrevida em 5 anos foi feita com o modelo de regressão de Cox, a curva de Kaplan-Meier e o teste Log-Rank. Utilizou-se o software R, versão 4.1.3 e o SAS 9.4. O nível de significância estatística adotado no estudo foi de 5%.

## Resultados

**Figura 1:**
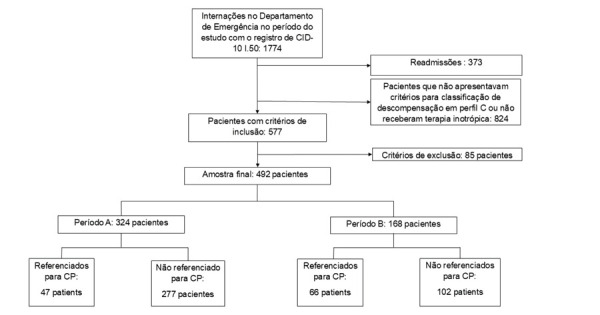
– Fluxograma de formação da amostra. CID-10: Classificação Internacional de Doenças, 10ª revisão; CP: cuidados paliativos.

Foram avaliadas 1774 admissões hospitalares no período do estudo, sendo 492 pacientes incluídos para análise final de dados, como demonstrado na figura 1. A Tabela 1 apresenta o perfil clínico-demográfico desses pacientes.

**Tabela 1:** – Perfil clínico-demográfico da população do estudo

Variáveis	Valores N=492
**Gênero masculino**	329 (66,9%)
Idade*	63,0 [52,0-72,0]
**Etiologia**	
Isquêmica	159 (32,3%)
Cardiomiopatia dilatada	98 (19,9%)
Doença de Chagas	83 (16,9%)
Valvar	68 (13,8%)
Hipertensiva	37 (7,5%)
Outras	76 (15,4%)
**Antecedentes pessoais**	
Hipertensão arterial sistêmica	271 (55,1%)
Dislipidemia	263 (53,5%)
Diabetes Mellitus	161 (32,7%)
Doença Renal Crônica	154 (31,3%)
Infarto do Miocárdio	113 (23,0%)
Eventos cerebrovasculares	84 (17,1%)
Cirurgia cardíaca prévia	113 (23,0%)
Ressincronizador cardíaco	48 (9,8%)
Cardioversor desfibrilador implantável	38 (7,7%)
**Medicamentos de uso prévio**	
IECA	221 (45,0%)
BRA	91 (18,5%)
Betabloqueador	432 (88,0%)
Espironolactona	291 (59,3%)
Digoxina	126 (25,7%)
Furosemida	411 (83,7%)
Fração de Ejeção (%)*	26,0 [22,0-33,0]
**Hospitalizações em 12 meses ou visitas ao pronto-socorro em 6 meses**	343 (69,7%)
**Classe funcional NYHA III ou IV**	356 (72,5%)

Fonte: autor. *mediana [IQ25-IQ75]. BRA: bloqueador do receptor de angiotensina II; IECA: inibidor da enzima conversora de angiotensina. NYHA: New York Heart Association.

A Tabela 2 apresenta as características da internação e tratamentos instituídos. As principais causas atribuídas à descompensação da IC foram: infecção (42,9%), progressão de doença (39,2%) e má aderência terapêutica (14,0%). A aplicação do escore ADHERE à admissão revelou que 52,0% dos pacientes eram de risco intermediário e 37,4% de risco elevado.

**Tabela 2:** – Caracterização da internação hospitalar

Variáveis	Valores (N=492)
Dias de internação hospitalar*	22,0 [13,0-42,0]
**Pacientes internados em UTI**	276 (56,1%)
Dias em UTI *	16,0 [9,00-34,4]
**Complicações na internação**	
Insuficiência Renal Aguda	414 (84,1%)
Infecção Nosocomial	215 (43,7%)
Delirium	134 (27,2%)
PCR reanimada	81 (16,5%)
**Uso de recursos hospitalares**	
Vasopressor^†^	183 (37,2%)
Ventilação mecânica de urgência	118 (24,0%)
Hemodiálise	84 (17,1%)
Balão intra-aórtico	62 (12,6%)
**Terapia inotrópica**	
Dias em uso de inotrópico*	13,0 [7,00-28,3]
Percentual de dias em uso de inotrópico*	72,6 [48,1-100]
Uso da dose máxima de inotrópico	183 (37,2%)
Uso combinado de inotrópicos	89 (18,1%)
**Cirurgia cardíaca**	
Transplante cardíaco	38 (7,7%)
Outra cirurgia cardíaca	9 (1,8%)
**Dispositivo de assistência circulatória mecânica**	
ECMO	3 (0,6%)
DACM de longa duração	2 (0,4%)
**Implante de DCEI**	16 (3,3%)
**Procedimentos percutâneos**	13 (2,6%)
**Alguma limitação de suporte terapêutico^**‡**^**	144 (29,3%)
**Decisão terapêutica tomada em conjunto com paciente**	23 (16,0%)
**Óbito na internação**	210 (42,7%)

Fonte: autor. DACM: dispositivos de assistência circulatória mecânica; DCEI: dispositivo cardíaco elétrico implantável ECMO: oxigenação por membrana extracorpórea; PCR: parada cardiorrespiratória; UTI: unidade de terapia intensiva. *mediana [IQ25-IQ75], ^†^exceto uso perioperatório, ^‡^Limitação de realização de reanimação cardiopulmonar, ou ventilação mecânica ou hemodiálise ou vasopressor.

### Interconsulta aos cuidados paliativos

**Figura 2:**
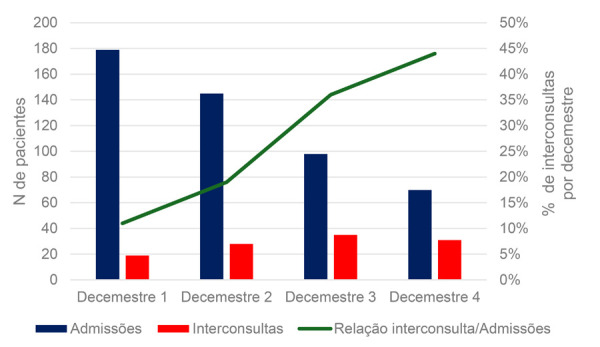
– Proporção de admissões hospitalares e de solicitações de interconsulta à equipe de cuidados paliativos a cada decemestre. Fonte: Autor.

Foram solicitadas 113 interconsultas à equipe de CP (23%). Apesar da redução nas internações na segunda metade do estudo, devido à mudança ocorrida no serviço neste período, para um modelo de admissão referenciada na unidade de emergência, se observou um aumento progressivo na taxa de solicitação de interconsulta para a equipe de CP a cada decemestre: 11%, 19%, 36% e 44% (Figura 2). A comparação da primeira e a segunda metade do estudo revela que esse aumento passou de 14,5% para 39,3% (p<0,01). Inversamente, nesse período houve uma redução do tempo necessário para o chamado, que passou de 10 dias (IQ 4,5-30) para 4,5 dias (IQ 1,0-15), (p<0,01).

Entre os pacientes encaminhados para CP, 66% eram do gênero masculino, tinham mediana de idade de 71 anos (IQ 62–79) e de fração de ejeção de 30% (IQ 24–35). Além disso, 83% apresentavam classe funcional NYHA III ou IV previamente à internação e 87% apresentavam risco ADHERE intermediário ou elevado à admissão. O pronto-socorro foi o setor que mais solicitou interconsultas (48%). Entre os pacientes internados em unidades de terapia intensiva (UTI), somente 14% foram referenciados para CP. No momento do chamado, poucos pacientes estavam em ventilação mecânica, hemodiálise, balão intra-aórtico ou em uso de vasopressores. Em contrapartida, 70% recebiam terapia inotrópica no momento da interconsulta.

As solicitações de interconsulta foram feitas com uma mediana de 8 dias (IQ 4-20) antes do óbito. A equipe de interconsulta de CP não conseguiu atender à solicitação de avaliação de 10% dos pacientes, pois o pedido foi feito nas últimas 48 horas de vida, incluindo quatro solicitações realizadas no mesmo dia do óbito.

A Tabela 3 apresenta a probabilidade de encaminhamento para CP, de acordo com critérios de IC avançada e marcadores de gravidade da internação.

**Tabela 3:** – Probabilidade de referenciamento para especialistas em cuidados paliativos

Variáveis clínicas	Modelo bruto				Modelo ajustado			
	RP	IC 95%		Valor p	RP	IC 95%		Valor p
Idade *	1,04	1,03	1,05	<0,01	1,02	1,01	1,04	<0,01
FE ≤ 30%	0,84	0,60	1,19	0,33	0,84	0,61	1,14	0,26
≥1 internação nos últimos 12 meses	0,78	0,56	1,08	0,13	0,74	0,55	0,99	0,04
Classe funcional NYHA III ou IV	1,88	1,19	2,95	<0,01	1,38	0,85	2,22	0,19
Não uso prévio de TMDD	0,65	0,37	1,13	0,13	0,81	0,57	1,16	0,26
IRA na internação	2,47	1,26	4,87	<0,01	1,13	0,56	2,28	0,73
PCR reanimada na internação	1,66	0,96	2,87	0,07	2,18	1,24	3,86	<0,01
% de dias em uso de inotrópico *	1,02	1,02	1,03	<0,01	1,02	1,01	1,03	<0,01
Uso combinado de inotrópicos	1,03	0,68	1,56	0,88	0,99	0,64	1,52	0,96
Dose máxima de inotrópico	1,78	1,29	2,45	<0,01	1,63	1,17	2,28	<0,01
Ausência de proposta de intervenção para IC avançada	2,01	1,38	2,93	<0,01	1,44	0,90	2,29	0,13

Fonte: Autor. *A cada aumento de um ponto percentual. IC: insuficiência cardíaca; IRA: insuficiência renal aguda; FE: fração de ejeção; NYHA: New York Heart Association; PCR: parada cardiorespiratória; TMDD: Terapia Medicamentosa Direcionada por Diretrizes.

A equipe de CP realizou ajustes das medidas sintomáticas, fez reuniões familiares e recomendou a transferência para a enfermaria de CP em 65%, 62% e 56% dos casos avaliados, respectivamente. A Tabela 4 apresenta as diferenças nas formas de abordagem de pacientes que foram encaminhados para CP e os atendidos apenas por cardiologistas.

**Tabela 4:** – Diferenciais da ação da equipe de cuidados paliativos

	Pacientes não encaminhados para CP N= 379	Pacientes encaminhados para CP N = 113	Valor-p
Tomada de decisão envolvendo o paciente	2 (0,52%)	21 (18,6%)	p <0,01
Uso de opioide para manejo sintomático na data do óbito *	15 (12,9%)	62 (65,9%)	p <0,01
Encaminhados para ambulatório de CP após alta hospitalar^†^	0 (0%)	13 (68,4%)	p <0,01

Fonte: Autor. CP: cuidados paliativos. * proporção referente ao número de óbitos: 116 no grupo não encaminhado e 94 no grupo encaminhado. ^†^proporção referente ao número de altas: 263 no grupo não encaminhado e 19 no grupo encaminhado.

Na Tabela 5 são comparados os grupos de pacientes encaminhados para CP e os que receberam tratamento convencional para IC avançada e o suporte hemodinâmico utilizado durante a internação. No caso dos dois pacientes que estiveram em lista para transplante cardíaco e foram avaliados pela equipe de CP, esse referenciamento ocorreu apenas após a sua inativação em lista.

**Tabela 5:** – Utilização de terapias para IC avançada e suporte ao choque durante a internação

	Pacientes não encaminhados para CP N= 379	Pacientes encaminhados para CP N = 113	Valor-p
Discussão sobre transplante cardíaco	144 (38,0%)	29 (25,7%)	0,02
Paciente que entraram em lista para transplante cardíaco	60 (15,8%)	2 (1,8%)	<0,01
Transplante cardíaco	38 (10,0%)	0 (0%)	<0,01
Vasopressor*	135 (35,6%)	48 (42,5%)	0,18
Implante de DCEI^†^	15 (3,9%)	1 (0,9%)	0,10
Suporte ventricular mecânico^‡^	59 (15,5%)	8 (2,1%)	0,02

Fonte: autor. CP: cuidados paliativos; DCEI: dispositivos cardíacos eletrônicos implantáveis. * exceto uso perioperatório, ^†^ressincronizador cardíaco ou cardioversor desfibrilador implantável, ^‡^balão intra-aórtico, ECMO ou DACM.

### Desfechos da internação e seguimento

**Figura 3:**
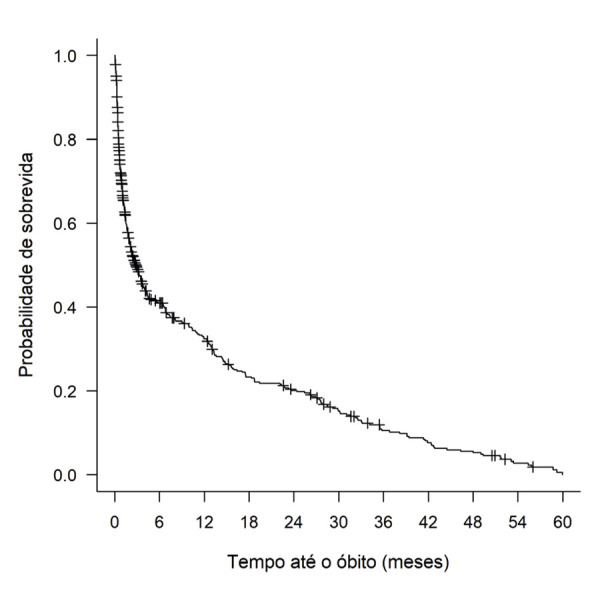
– Probabilidade de sobrevida da população geral em 5 anos. Fonte: Autor.

Observou-se que tanto a mortalidade hospitalar, quanto em 5 anos foram elevadas, 43% e 80%, respectivamente. Entre os pacientes que receberam alta, 49% foram readmitidos pelo menos uma vez nos anos subsequentes. O encaminhamento para o ambulatório de CP ocorreu apenas nos pacientes que foram atendidos pelos CP na internação. Dos 394 óbitos, 67% ocorreram nos primeiros 6 meses após a admissão inicial (Figura 3).

## Discussão

Este estudo é o maior registro brasileiro de pacientes com IC avançada internados com necessidade de terapia inotrópica, sendo inédito ao propor a investigação da abordagem de CP nessa população. A gravidade e a complexidade dessa população fazem das intervenções paliativas algo premente.^23,25-27^

A análise de pacientes internados em choque cardiogênico, em 2020, do *National Inpatient Sample Database,*^27^ observou uma taxa de encaminhamento para CP semelhante à encontrada em neste estudo (22% x 23%). Os autores consideraram que essa baixa taxa reflete o quão pouco os CP são integrados na da cardiologia, sendo reservados apenas àqueles com alta probabilidade de morte.

O aumento progressivo na frequência de encaminhamentos, observado ao longo dos 40 meses de estudo, pode ser explicado, em parte, pelo efeito multiplicador das ações da equipe de CP. Cada vez mais integradas nas rotinas hospitalares, como algo habitual, a educação de profissionais e a divulgação das práticas de CP auxiliam na desmistificação de conceitos equivocados, favorecendo a integração.

Um estudo com pacientes em choque cardiogênico que requeriam suporte circulatório mecânico observou um aumento de 9,4% para 16,8%, em seis anos, na taxa de referenciamento para CP. Os autores atribuíram esse achado ao crescente reconhecimento entre os profissionais de saúde, quanto a importância dos CP para pacientes com IC na melhoria da qualidade de vida, associado às recomendações das diretrizes de sociedades de cardiologia.^28^

Apesar desse aumento crescente na taxa de referenciamento para CP, se estima que ainda há uma demanda reprimida. Este estudo buscou identificar oportunidades para facilitar e otimizar a integração dos CP na prática cardiológica.

Observou-se que não há uma padronização de critérios utilizados pelos cardiologistas para referenciar seus pacientes para CP. Marcadores de IC avançada que são associados a um pior prognóstico, como fração de ejeção muito reduzida, classe funcional NYHA III ou IV e hospitalizações recorrentes por IC, prevalentes neste estudo, não foram associados à maior probabilidade de referenciamento. Dois estudos mostraram que a padronização de critérios se mostrou mais efetiva do que aguardar a iniciativa dos cardiologistas, aumentando a taxa de referenciamento para CP, de 6,4% para 20%^29^ e de 28% para 46%.^30^ Critérios claros que orientem cardiologistas a identificar pacientes que se beneficiam de CP facilitam essa integração.^22^

Neste estudo, pacientes que permaneceram maior tempo em uso de terapia inotrópica ou que necessitaram de dose máxima de inotrópico foram mais referenciados para CP, possivelmente por representar uma doença de mais difícil controle. Marcadores hospitalares de gravidade, como internação em UTI, disfunções orgânicas secundárias e maior necessidade de suporte hemodinâmico, como vasopressores ou suporte ventricular mecânico,^31^ são critérios para o encaminhamento para CP.^20-22^

Poucos pacientes internados em UTI, neste estudo, foram referenciados para CP, sendo que apenas 15,5% dos óbitos ocorridos nesses setores contaram com o acompanhamento da equipe de CP. As limitações de suporte terapêutico foram decididas tardiamente, com uma mediana de quatro dias antes do óbito. Dados de literatura corroboram essa prática, sendo observada uma taxa de encaminhamento para CP em pacientes com IC internados em UTI variando entre 1,2% e 4,1%^32^ e as definições de limitação de suporte terapêutico sendo tomadas nas últimas 48 horas de vida.^7,16^

Essa forma dicotômica de abordagem da doença, reservando os CP exclusivamente para o final de vida, excluindo-o de fases em que ainda há perspectivas de tratamentos modificadores de doença, é uma das principais barreiras para a sua integração precoce.^7,16,18,22,29^

Destaca-se que neste estudo, as terapêuticas que têm a capacidade de modificar a evolução da IC na sua fase avançada, como transplante cardíaco e os DACM, foram pouco realizadas nesta amostra, limitadas pela baixa disponibilidade de dispositivos, órgãos para transplante ou por contraindicações clínicas à sua realização.

Nenhum paciente submetido a transplante cardíaco ou DACM foi encaminhado para CP, apesar de estarem em um serviço que oferece essa avaliação a qualquer paciente, independentemente da etiologia, prognóstico ou possibilidades terapêuticas.

Apesar do reconhecimento da importância da integração dos CP para pacientes listados para transplante cardíaco,^33^ isso não foi observado nesta coorte. Isso contraria as diretrizes da *International Society of Heart and Lung Transplantation*, que recomendam que os CP sejam parte integral do tratamento da IC, realizados juntamente com a avaliação de terapias avançadas, ao longo da evolução e nos cuidados de fim de vida, com o objetivo de aliviar os sintomas, auxiliar na definição de metas terapêuticas e proporcionar um atendimento amplo às preferências individuais.^34,35^

Outra diferença significativa na forma de abordagem da equipe de CP foi o maior envolvimento dos pacientes nas discussões para a tomada de decisão (18,6% x 0,52%, p<0,01). Sidebottom et al., mostraram que pacientes encaminhados para CP apresentam chance 2,87 vezes maior de estabelecer um planejamento avançado de cuidados do que aqueles atendidos apenas por cardiologistas (p=0,03).^25^ A comunicação adequada com pacientes e familiares permite o alinhamento das estratégias terapêuticas aos seus valores, objetivos e preferências de cuidado.^9,15,28,36^

Neste estudo se observou que pacientes atendidos pela equipe de CP mais frequentemente receberam opioides no final da vida, em atenção aos seus sintomas, como dor e dispneia. Isso sugere um maior reconhecimento e abordagem dos sintomas quando são avaliados por especialistas em CP. De forma semelhante, Ye et al. observaram uma menor avaliação das necessidades paliativas quando a abordagem é realizada por não especialistas em CP. Justificam o achado pela subestimação de sua relevância ou por falta de treinamento e de conhecimentos em CP.^18^

Os inotrópicos são outra forma de manejar sintomas e proporcionar qualidade de vida em pacientes com IC avançada, à medida que buscam equilibrar a hemodinâmica. Com intuito paliativo, principalmente em pacientes não candidatos a terapias para IC avançada, é recomendado o envolvimento de especialistas em CP na condução do tratamento.^37^ Neste estudo, 91% dos pacientes transferidos para enfermaria de CP recebiam inotrópicos para conforto, sendo lá titulados para essa finalidade.

A enfermaria de CP, neste serviço, se propõe a realizar a compensação clínica enquanto alivia os sintomas, com perspectiva de alta hospitalar, e também serve como modelo *hospice,* com foco em oferecer conforto no final da vida. Foram desospitalizados 19% dos pacientes para lá encaminhados em uso de inotrópicos. Apenas 5% dos óbitos hospitalares ocorreram na enfermaria de CP.

Diop et al. observou que pacientes que receberam CP foram mais transferidos para *hospice* do que os que receberam tratamento convencional (34,8% x 18,35, respectivamente, p<0,001).^38^

Considerando que um dos critérios de elegibilidade para internação em *hospice*, segundo o *Medicare/Medicaid*, é uma expectativa de vida inferior a seis meses^19^ e que, no presente estudo, 67% dos óbitos ocorreram dentro desse período, sendo potencialmente elegíveis para cuidados em *hospice*, se pode supor que mais pacientes poderiam ter indicação de receber CP do que os que foram efetivamente referenciados.

Este estudo apresenta diversas limitações, principalmente relacionadas à sua metodologia retrospectiva, dependente dos registros médicos, que podem ser falhos, especialmente com relação às documentações de sintomas, valores pessoais e diretivas, nos pacientes não atendidos pela equipe de CP. Esses fatores limitam a mensuração da real demanda por CP na amostra. Além disso, a natureza unicêntrica do estudo é outro fator limitante, o que restringe o seu potencial de generalização. Por fim, não foram avaliadas as internações em outros serviços de pacientes que receberam alta hospitalar, o que subestimou a taxa de reinternações.

## Conclusão

Pacientes em descompensação de IC com necessidade de terapia inotrópica apresentam alta mortalidade hospitalar e no seguimento de cinco anos, o que reforça a necessidade da abordagem integrada de CP, de forma precoce, ainda pouco realizada na prática clínica da cardiologia. O encaminhamento tardio reduz os potenciais benefícios da abordagem paliativa. A utilização de marcadores de pior prognóstico, momentos críticos de tomada de decisão terapêutica, manejo de sintomas e a consideração de transferência para *hospice* são oportunidades de melhorar a integração de CP no tratamento da IC. É importante aprimorar a educação dos profissionais de saúde e desenvolver estratégias que favoreçam a sua implementação precoce, o que pode otimizar a integração entre cardiologia e CP, com o potencial de impactar positivamente na qualidade da assistência e em desfechos clínicos.
