# Supplementary material for: Cuidados Paliativos na Insuficiência Cardíaca Descompensada com Necessidade de Terapia Inotrópica: Oportunidades de Integração para Melhoria dos Desfechos
Source: Arq Bras Cardiol. 2026 Jan 9;122(12):e20250188. [Article in Portuguese] doi: 10.36660/abc.20250188 (PMC12978370; doi:10.36660/abc.20250188)
Supplement: Translation

# Translation: Palliative Care in Decompensated Heart Failure Requiring Inotropic Therapy: Opportunities for Integration to Improve Outcomes

**DOI:** 10.36660/abc.20250188i

**Authors:** Daniel Battacini Dei Santi, Mucio Tavares de Oliveira, Ricardo Tavares de Carvalho

**Affiliations:** 1 Brazil Instituto do Coração do Hospital das Clínicas da Faculdade de Medicina da Universidade de São Paulo, São Paulo, SP – Brazil; 2 Brazil Hospital das Clinicas da Faculdade de Medicina da Universidade de São Paulo – Cuidados Paliativos, São Paulo, SP – Brazil

**Keywords:** Heart Failure, Cardiogenic Shock, Palliative Care, Critical Illness, Hospice Care

## Abstract

**Background:**

Advanced heart failure (HF) is associated with high morbidity and mortality rates, compromising the functionality and quality of life of patients and their families. Hospitalizations exacerbate disease severity, particularly when inotropic therapy is required. Palliative care (PC) supports the management of suffering caused by severe illnesses but is infrequently utilized in cardiology.

**Objective:**

To evaluate the integration of PC in the management of decompensated HF, identifying opportunities to enhance patient care.

**Method:**

This unicentric, retrospective, observational study was conducted between February 2015 and May 2018 with HF patients undergoing inotropic therapy. The study analyzed referrals for PC, the approach adopted by the PC-consultation team, and patient outcomes, including an analysis of 5-year survival rates. Statistical significance level: 5%.

**Results:**

A total of 492 patients were included (66.9% male, median age 63 years, IQR 52-72). PC referral occurred in 23% of cases, with a median of 8.0 days (IQR 4.0-20) before death. Only 14% of intensive care patients were referred, and no transplant patients received PC evaluations. Patients assessed by the PC team were more involved in decision-making and received more opioid prescriptions for symptom management than those managed exclusively by cardiologists (p<0.01). In-hospital and 5-year mortality rates were 42% and 80%, respectively.

**Conclusions:**

Patients experiencing decompensated HF demonstrate high mortality rates and are rarely referred to PC, often in the final days of life, limiting the potential benefits of this approach. Enhanced medical education in PC and the development of strategies to promote its integration may improve patient outcomes.

## Introduction

Heart failure (HF) has a high mortality rate worldwide, accounting for over 9 million deaths annually.^1^ In Brazil, it is estimated that 2 million people are affected, with more than 240,000 new cases recorded each year.^2^ In 2019, HF caused over 196,000 hospitalizations, resulting in R$3 billion in healthcare expenditures.^3^

Hospitalization is often required when symptoms worsen, particularly in patients with congestive symptoms and low cardiac output, classified as “clinical hemodynamic profile C”, known as “cold and wet”.^4-6^ These cases demand extensive hospital support, prolonged stays, and intensive life-support interventions.^5,6^

Disease-modifying therapies for advanced HF are limited to a few strategies, such as heart transplantation and mechanical circulatory support devices (MCSDs), accessible to only a small proportion of patients.^7,8^ These individuals frequently experience severe symptoms that impair functionality and quality of life, necessitating complementary approaches to conventional treatment.^7,8^

Palliative care (PC) plays an important role in this context.^9,10^ According to Radbruch et al, it is an “active holistic care of individuals across all ages with serious health-related suffering due to severe illness and especially of those near the end of life. It aims to improve the quality of life of patients, their families, and their caregivers”.^11^

Recent guidelines from specialist societies have recommended the early integration of PC into the treatment of patients with HF,^12-14^ focusing on symptom control and advanced care planning.^9,11,15,16^ This approach can positively influence clinical outcomes, such as avoiding futile interventions and favoring transfer to hospice units, which prioritize comfort at the end of life.^9,10,17-19^

However, there are several barriers to the integration of PC in cardiology, including the lack of knowledge among health professionals and the misconception that PC is exclusively for end-of-life care, in the context of the absence of modifying therapeutic prospects for the disease.^20,21^ The unpredictability of the clinical course of HF, challenges in establishing a precise prognosis, and the lack of standardized referral criteria further complicate this integration.^22^ Changing this scenario requires structural measures, including expanded medical education, the development of clinical care protocols, and the strengthening of institutional incentives, to foster a cultural shift that enhances the quality of care.

Patients in decompensated HF requiring inotropic therapy are poorly studied in the literature, especially regarding the practice of PC.^23^ This study aims to investigate how PC specialists are integrated into the treatment of these patients, exploring opportunities to optimize care.

## Method

### Study design and population

This is a retrospective, single-center observational study conducted in a Brazilian quaternary teaching hospital, a reference center for HF treatment. The cohort consisted of patients admitted to the emergency department between February 1^st^, 2015, and May 31^st^, 2018, with a decompensated HF profile C, requiring inotropic therapy.

Patients were selected from hospitalization records with an ICD-10 code for HF (I.50). Their data were collected through electronic hospital records and transcribed into a protected spreadsheet for statistical analysis. The sample’s 5-year survival rate was then analyzed.

Patients with a previous diagnosis of HF were included, regardless of etiology, and required a documented by echocardiogram left ventricular ejection fraction of less than 50% or moderate or significant right ventricular dysfunction. Patients had to present clinical or laboratory criteria allowing classification in profile C, and inotropic therapy had to be initiated within the first 5 days of hospital admission.

Exclusion criteria included patients under the age of 18, those with de novo HF diagnosis, or those for whom acute coronary syndrome was the primary diagnostic hypothesis. Patients were also excluded if inotropic therapy duration was less than 24 hours, or because of anesthetic/surgical intervention, or if they were transferred from other services using inotropes or vasopressors. Additionally, patients whose initial electronic records did not allow the characterization of hospital admission or whose hospitalization ended outside the hospital complex were excluded. In cases of multiple admissions during the study period, only the first qualifying admission was considered.

### Variables of interest

Clinical and demographic characteristics, as well as emergency room admission details, were analyzed. Risk assessment was performed using the Acute Decompensated Heart Failure National Registry (ADHERE) tool.^24^ Hospitalization duration, treatments performed, and referral processes to the PC team were evaluated.

This analysis included the frequency of inter-consultation requests, referral times, characteristics of referred patients, contexts of PC consultation requests, and interventions performed by the PC team.

Patients receiving PC were compared to those managed exclusively by cardiologists regarding opioid use at end-of-life, patient involvement in decision-making, and PC outpatient referrals post-discharge.

### Statistical analysis

The data was described in absolute and relative frequencies. In order to compare PC-consultation requests over the 40-month study period, four 10-month intervals were defined. Continuous variables were presented as medians with 25% and 75% interquartile ranges (IQR). Group comparisons used the chi-square test. The Poisson regression model estimated the non-adjusted and adjusted prevalence ratios for binary outcomes.

The variables included in the multivariate regression were: age, ejection fraction, hospital admission within the previous 12 months, New York Heart Association (NYHA) functional class, prior use of guideline-directed medical therapy (GDMT), presence of renal dysfunction, cardiopulmonary arrest, inotropic therapy (duration of use, need for drug combination, or maximum dose requirement), and therapeutic strategies proposed for advanced HF during hospitalization.

Five-year survival probability was analyzed using the Cox regression model, Kaplan-Meier curves, and the Log-Rank test. Statistical analyses were conducted using R software (version 4.1.3) and SAS (version 9.4). A statistical significance level of 5% was adopted.

## Results

A total of 1,774 hospital admissions were assessed during the study period, with 492 patients included for final data analysis, as presented in Figure 1. Table 1 shows the clinical and demographic profile of these patients.

**Figure 1:** – Study sample flowchart. ICD: International Classification of Diseases, 10th Revision; PC: palliative care. Source: Author.

**Table 1:** – Clinical and demographic characteristics of the study population

Variables	Values N=492
**Male gender**	329 (66.9%)
Age*	63.0 [52.0-72.0]
**Heart Failure Etiology**	
Ischemic	159 (32.3%)
Dilated cardiomyopathy	98 (19.9%)
Chagas disease	83 (16.9%)
Valvular	68 (13.8%)
Hypertensive	37 (7.5%)
Others	76 (15.4%)
**Medical History**	
Systemic arterial hypertension	271 (55.1%)
Dyslipidemia	263 (53.5%)
Diabetes Mellitus	161 (32.7%)
Chronic kidney disease	154 (31.3%)
Myocardial infarction	113 (23.0%)
Cerebrovascular events	84 (17.1%)
Previous cardiac surgery	113 (23.0%)
Cardiac resynchronization therapy	48 (9.8%)
Implantable cardioverter-defibrillator	38 (7.7%)
**Previous Medications**	
ACEI	221 (45.0%)
Angiotensin receptor blocker	91 (18.5%)
Beta-blocker	432 (88.0%)
Spironolactone	291 (59.3%)
Digoxin	126 (25.7%)
Furosemide	411 (83.7%)
Ejection Fraction (%)*	26.0 [22.0-33.0]
**Hospitalizations in the past 12 months or emergency department visits in the past 6 months**	343 (69.7%)
**NYHA functional class III or IV**	356 (72.5%)

Source: Author. *median [IQR 25-75]. ACEI: Angiotensin-converting enzyme inhibitor; NYHA: New York Heart Association.

Table 2 shows the characteristics of hospitalization and treatment performed. The main causes attributed to HF decompensation were infections (42.9%), disease progression (39.2%), and poor therapeutic adherence (14.0%). Applying the ADHERE score on admission revealed that 52.0% of patients were at intermediate risk and 37.4% at high risk.

**Table 2:** – Hospitalization characteristics

Variables	Values (N=492)
Days of hospital stay*	22.0 [13.0-42.0]
**Patients admitted to the ICU**	276 (56.1%)
Days in ICU*	16.0 [9.00-34.4]
**Hospitalization Complications**	
Acute kidney injury	414 (84.1%)
Nosocomial infection	215 (43.7%)
Delirium	134 (27.2%)
Resuscitated cardiac arrest	81 (16.5%)
**Use of Hospital Resources**	
Vasopressor ^†^	183 (37.2%)
Emergency mechanical ventilation	118 (24.0%)
Hemodialysis	84 (17.1%)
Intra-aortic balloon pump	62 (12.6%)
**Inotropic Therapy**	
Days on inotropic therapy*	13.0 [7.00-28.3]
Percentage of days on inotropic therapy*	72.6 [48.1-100]
Use of maximum inotropic dose	183 (37.2%)
Combined use of inotropes	89 (18.1%)
**Cardiac Surgery**	
Heart transplant	38 (7.7%)
Other cardiac surgery	9 (1.8%)
**Mechanical Circulatory Support Devices**	
ECMO	3 (0.6%)
Long-term MCSD	2 (0.4%)
**Implantable cardiac electronic device**	16 (3.3%)
**Percutaneous procedures**	13 (2.6%)
**Withholding or withdrawing life-sustaining therapies ^**‡**^**	144 (29.3%)
**Therapeutic decision-making involving the patient**	23 (16.0%)
**In-hospital mortality**	210 (42.7%)

Source: Author. MCSD: Mechanical circulatory support devices, ECMO: Extracorporeal membrane oxygenation; ICU: Intensive care unit. *median [IQR 25-75]. ^†^Except for perioperative use; ^‡^Decisions involving cardiopulmonary resuscitation orders, mechanical ventilation, hemodialysis, or vasopressor therapy.

### Palliative care consultation

A total of 113 consultations were requested of the PC team (23%). Despite the reduction in hospital admissions during the second half of the study, due to changes implemented in the service during this period, shifting to a referral-based admission model in the emergency unit, there was a progressive increase in the PC referral rate every ten months: 11%, 19%, 36% and 44% (Figure 2). A comparison between the first and second halves of the study shows that this increase rose from 14.5% to 39.3% (p<0.01). Conversely, during this period, there was a reduction in the time required for the request, which decreased from 10 days (IQ 4.5-30) to 4.5 days (IQ 1.0-15) (p<0.01).

**Figure 2:** – Proportion of hospital admissions and PC consultation requests per ten-month period. PC: palliative care. Source: Author.

Among the patients referred to PC, 66% were male, had a median age of 71 years (IQR 62-79), and an ejection fraction of 30% (IQR 24-35). Additionally, 83% had NYHA functional class III or IV before admission, and 87% were at intermediate or high risk on admission (ADHERE). The emergency department was the facility that requested the most PC consultations (48%). Among patients admitted to intensive care units (ICUs), only 14% were referred to PC. At the time of the request, few patients were on mechanical ventilation, hemodialysis, intra-aortic balloon, or using vasopressors. Conversely, 70% were receiving inotropic therapy at the time of the consultation request.

PC-consultation requests were made a median of 8 days (IQ 4-20) before death. The PC consultation team was unable to fulfill the evaluation request for 10% of the patients because the request was made within the last 48 hours of life, including four requests made on the same day of the death.

Table 3 shows the probability of referral for PC according to advanced HF criteria and hospitalization severity markers.

**Table 3:** – Probability of referral to PC specialists based on patient characteristics and severity markers

Clinical Variables	Non-adjusted model				Adjusted model			
	PR	CI 95%		p-value	PR	CI 95%		p-value
Age *	1.04	1.03	1.05	<0.01	1.02	1.01	1.04	<0.01
Ejection Fraction < 30%	0.84	0.60	1.19	0.33	0.84	0.61	1.14	0.26
≥1 hospitalization in the past 12 months	0.78	0.56	1.08	0.13	0.74	0.55	0.99	0.04
NYHA functional class III / IV	1.88	1.19	2.95	<0.01	1.38	0.85	2.22	0.19
No prior use of GDMT	0.65	0.37	1.13	0.13	0.81	0.57	1.16	0.26
AKI during hospitalization	2.47	1.26	4.87	<0.01	1.13	0.56	2.28	0.73
Resuscitated CPA during hospitalization	1.66	0.96	2.87	0.07	2.18	1.24	3.86	<0.01
% of days on inotropic therapy*	1.02	1.02	1.03	<0.01	1.02	1.01	1.03	<0.01
Combined use of inotropes	1.03	0.68	1.56	0.88	0.99	0.64	1.52	0.96
Maximum inotropic dose	1.78	1.29	2.45	<0.01	1.63	1.17	2.28	<0.01
Absence of proposed intervention for advanced HF	2.01	1.38	2.93	<0.01	1.44	0.90	2.29	0.13

Source: Author. * For each one-percentage-point increase. CI: confidence interval; AKI: acute kidney injury; CPA: cardiopulmonary arrest; GDMT: guideline-directed medical therapy; HF: heart failure; NYHA: New York Heart Association; PR: prevalence ratio.

The PC team adjusted symptomatic measures, held family meetings, and recommended transfer to the PC ward in 65%, 62% and 56% of the cases assessed, respectively. Table 4 shows the differences in the approach to patients who were referred to PC and those evaluated only by cardiologists.

**Table 4:** – Differences in PC team approach

	Patients not referred to PC N= 379	Patients referred to PC N = 113	p-value
Decision-making involving the patient	2 (0.52%)	21 (18.6%)	p <0.01
Opioid use for symptomatic management on the date of death*	15 (12.9%)	62 (65.9%)	p <0.01
Referred to outpatient PC after hospital discharge^†^	0 (0%)	13 (68.4%)	p <0.01

Source: Author. PC: Palliative care. *Relative to the number of deaths: 116 in the non-referred group and 94 in the referred group. ^†^Relative to the number of discharges: 263 in the non-referred group and 19 in the referred group.

Table 5 compares the groups of patients referred to PC and those who received conventional treatment for advanced HF and the hemodynamic support used during hospitalization. In the case of the two patients who were on the list for heart transplantation and were assessed by the PC team, this referral only took place after their removal from the list.

**Table 5:** – Utilization of advanced HF therapies and shock support during hospitalization

	Patients not referred to PC N= 379	Patients referred to PC N = 113	p-value
Heart transplantation discussion	144 (38.0%)	29 (25.7%)	0.02
Patients listed for heart transplantation	60 (15.8%)	2 (1.8%)	<0.01
Heart transplantation	38 (10.0%)	0 (0%)	<0.01
Vasopressor*	135 (35.6%)	48 (42.5%)	0.18
Implantation of CIED^†^	15 (3.9%)	1 (0.9%)	0.10
Ventricular mechanical support^‡^	59 (15.5%)	8 (2.1%)	0.02

Source: Author. CIED: Cardiac Implantable Electronic Device; PC: Palliative Care. *Except perioperative use. ^†^Cardiac resynchronization therapy or implantable cardioverter-defibrillator. ^‡^Intra-aortic balloon pump, Extracorporeal Membrane Oxygenation or long term Mechanical Circulatory Support Device.

### Hospitalization and follow-up outcomes

Both in-hospital and 5-year mortality rates were high, at 43% and 80%, respectively. Among the patients who were discharged, 49% were readmitted at least once in subsequent years. Referral to the PC outpatient clinic only occurred in patients who had been evaluated by the PC specialists during hospitalization. Of the 394 deaths, 67% occurred in the first 6 months after initial admission (Figure 3).

**Figure 3:** – Five-Year Survival Probability of the Study Population. Source: Author.

## Discussion

This study is the largest Brazilian registry of patients with advanced HF hospitalized requiring inotropic therapy and is unprecedented in proposing to investigate the PC approach in this population. The severity and complexity of this population make palliative interventions an urgent issue.^23,25-27^

An analysis of patients hospitalized in cardiogenic shock in 2020 from the National Inpatient Sample Database registry,^27^ found a referral rate for PC similar to that observed in this study (22% x 23%). The authors considered that this low rate reflects how poorly PC is integrated into cardiology, with referrals being reserved for those with a high probability of death.

The observed increase in referral frequency over the 40 months of the study may be partly explained by the progressive influence of the PC team’s actions. As PC became increasingly integrated into hospital routines, professional education and the dissemination of PC practices contributed to dispelling misconceptions, thereby facilitating integration.

A study of patients in cardiogenic shock requiring mechanical circulatory support reported an increase in PC referral rates from 9.4% to 16.8% over six years. The authors attribute this finding to the growing recognition among health professionals of PC’s importance in improving quality of life for patients with HF, alongside recommendations from cardiology society guidelines.^28^

Despite the increase in PC referral rates, it is estimated that there is still a significant unmet demand. This study aimed to identify opportunities to facilitate and optimize the integration of PC into cardiology practice.

It was observed that there is no standardization of the criteria used by cardiologists in referring patients for PC. Markers of advanced HF that are associated with poor prognosis, such as very low ejection fraction, NYHA functional class III or IV, and recurrent hospitalizations for HF, which were prevalent in this study, did not correlate with a higher likelihood of referral. Two studies demonstrated that the standardization of criteria proved more effective than waiting for cardiologists’ initiative, increasing the PC referral rate from 6.4% to 20%^29^ and from 28% to 46%.^30^ Clearer criteria to help cardiologists identify patients who would benefit from PC can also enhance integration.^22^

In this study, patients on prolonged inotropic therapy or requiring maximum doses were more likely to be referred to PC, possibly due to the complexity of their condition, which represents a more challenging disease. Hospital severity markers, such as ICU admission, secondary organ dysfunction, and increased need for hemodynamic support (e.g., vasopressors or mechanical ventricular support),^31^ are also considered PC referral criteria.^20-22^

Few ICU patients in this study were referred to PC, and only 15.5% of deaths in these settings were followed by the PC team. Decisions regarding withholding and withdrawing therapeutic support were often delayed, with a median of four days before death. Literature data corroborate this trend, with PC referral rates for ICU HF patients ranging from 1.2% to 4.1%^32^ and therapeutic limitation decisions occurring within the last 48 hours of life.^7,16^

This dichotomous way of approaching the disease, which reserves PC exclusively for end-of-life stages and excludes it from phases when disease-modifying treatments are still viable, is a major barrier to early integration.^7,16,18,22,29^ Notably, no patients undergoing heart transplantation or MCSDs were referred to PC in this study.

It is noteworthy that, in this study, therapies capable of modifying the course of advanced heart failure, such as heart transplantation and MCSDs, were infrequently performed in this cohort, mainly due to limited availability of devices or donor organs, or clinical contraindications to their use.

None of the patients who underwent heart transplantation or MCSD implantation were referred to PC, despite being in a center where such evaluation is available to all patients, regardless of disease etiology, prognosis, or therapeutic possibilities.

Despite recognition of the value of integrating PC to patients listed for heart transplantation, this was not observed in this cohort.^33^ This finding contradicts the guidelines of the International Society of Heart and Lung Transplantation, which recommend that PC should be an integral component of HF treatment, conducted alongside evaluations for advanced therapies, including heart transplantation and MCSDs. PC should also be involved at the end of life to alleviate symptoms, assist in defining therapeutic goals, and address individual preferences comprehensively.^34,35^

A notable difference in the PC team’s approach was the greater involvement of patients in decision-making discussions (18.6% vs. 0.52%, p<0.01). Sidebottom et al. demonstrated that patients referred to PC were 2.87 times more likely to engage in advanced care planning than those managed exclusively by cardiologists (p=0.03).^25^ Effective communication with patients and families enables therapeutic strategies to align with their values, goals, and care preferences.^9,15,28,36^

This study found that patients evaluated by the PC team more frequently received opioid prescriptions at the end of life to manage symptoms such as pain and dyspnea. This suggests that PC specialists are better at recognizing and addressing symptom management. Similarly, Ye et al. observed that PC needs were less frequently assessed when evaluations were conducted by non-specialists, attributing this to either underestimation of symptom relevance or a lack of PC training among cardiologists.^18^

Inotropic drugs are another strategy for symptom management and improving quality of life in patients with advanced HF, particularly for those ineligible for advanced HF therapies, as they aim to balance hemodynamics. In such cases, PC specialist involvement is recommended.^37^ In this study, 91% of the patients transferred to the PC ward were receiving inotropes for comfort, with doses titrated accordingly.

In this service, the PC ward serves both for clinical stabilization while implementing measures for symptom relief, with the possibility of discharge, and as a hospice care model, focused on end-of-life comfort. A total of 19% of the patients referred to the PC ward on inotropes were discharged. Only 5% of hospital deaths occurred in the PC ward.

Diop et al. found that patients receiving PC were more frequently transferred to hospice compared to those receiving conventional treatment (34.8% vs. 18.3%, p<0.001).^38^

Considering that one eligibility criterion for hospice admission, according to Medicare/Medicaid, is a life expectancy of less than six months^19^ and that 67% of the deaths in this study occurred within this period, potentially eligible for hospice care, it can be assumed that more patients would have been indicated to PC consultation than those who were actually referred.

This study has several limitations, mostly related to its retrospective design, which depends on medical records, which may be incomplete, especially regarding documentation of symptoms, personal values, and directives for patients not assessed by the PC team. These factors limit the accurate measurement of the real demand for PC in the sample. Additionally, the single-center nature of the study restricts the generalizability of the findings. Finally, post-discharge admissions to other services were not assessed, potentially underestimating hospital readmission rates.

## Conclusion

Patients with decompensated HF requiring inotropic therapy have high in-hospital and follow-up mortality, reinforcing the need for an integrated and early approach by PC, which is still rarely implemented in clinical cardiology practice. Late referral diminishes the potential benefits of the palliative approach. Utilizing markers of poor prognosis, addressing critical moments in therapeutic decision-making, managing symptoms effectively, and considering hospice transfer are key opportunities to enhance PC integration in HF treatment. Optimizing integration requires improved education for health professionals and the development of strategies that encourage early PC implementation, which can positively impact the quality of care and clinical outcomes.
